# Changes in Pitch Velocity and Selection in Major League Baseball Pitchers Following the Ulnar Collateral Ligament Reconstruction

**DOI:** 10.7759/cureus.62551

**Published:** 2024-06-17

**Authors:** Oluwa Toba J Akinleye, Jason B Kreinces, Bruno Alonso, Daniel Bleykhman, Daniel Zelazny

**Affiliations:** 1 Orthopaedic Surgery, New York Medical College, New York City, USA; 2 Orthopaedic Surgery, Westchester Medical Center, New York City, USA

**Keywords:** baseball pitcher, professional baseball players, surgery, ulnar collateral ligament, sports overuse injuries

## Abstract

Background: The ulnar collateral ligament (UCL) is a soft-tissue stabilizer of the elbow, that is commonly injured among baseball pitchers due to excess valgus stress in overhead throwing motions. The location of a UCL tear typically ranges from the central aspect of the ligament to an avulsion-type injury at its proximal insertion site on the medial epicondyle of the humerus, or its distal insertion site on the ulna. The effect of UCL reconstruction on pitching performance has become a well-studied topic in medical literature. In our study, we aimed to identify general performance patterns amongst those having undergone UCL reconstruction surgery.

Methods: Data for patients with UCL reconstruction performed between 2010 and 2020 were extracted from publicly available databases. Pitching data was extracted from Brooks Baseball, a publicly available database for Major League Baseball (MLB) player statistics. We identified patients who played at least one full season after surgery and rehabilitation. Patient characteristics were evaluated for intergroup differences.

Results: Of 109 patients with UCL reconstruction, 87 were included in the final analysis. Compared to the preoperative group, the average postoperative fastball usage rate was less despite there being an increase in the off-speed usage rate. Velocity analysis demonstrated shifts of less than 1% for all three pitch groups compared to preoperative velocity average values (all P-values <0.05). Earned Run Average (ERA)+ demonstrates a decrease in the postoperative group; this finding was not significant (P=0.61).

Conclusions: Patients treated surgically demonstrated a throwing tendency for their secondary and tertiary pitches and a decreased usage of fastballs. Further studies are needed to explore the factors contributing to the change in pitching performance.

## Introduction

Ulnar collateral ligament (UCL) reconstruction is a common surgery among pitchers in Major League Baseball (MLB). The UCL is a crucial soft-tissue stabilizer of the elbow during overhead throwing [1]. Specifically, its role during the overhead throwing motion is to protect the elbow against excess valgus stress [1]. This is especially important in MLB players given the large amount of valgus torque placed on the elbow with increasing velocities seen in the game of baseball today [1,2]. UCL tears in MLB pitchers commonly occur in the anterior bundle of the ligament, which is the primary valgus stabilizer from 30° to 90° of flexion [1]. Injuries to the UCL can occur either chronically through degeneration over time or acutely [3]. The location of a UCL tear varies from the central aspect of the ligament to an avulsion-type injury from its proximal insertion site on the medial epicondyle of the humerus or its distal insertion site on the ulna [3].

There are a variety of studies in the sports medicine literature that have analyzed the effect of UCL reconstruction on pitching performance in MLB and amateur pitchers as well as potential risk factors for UCL injury leading up to the procedure [4-7]. Reportedly the mean release point of pitchers requiring UCL reconstruction is more lateral than in pitchers who do not require surgery, which may suggest that a more lateral pitch release angle is a predictor for UCL injury [4]. McKnight [6] conducted a study of 56 MLB pitchers to evaluate pitch accuracy, velocity, and movement following surgical reconstruction. They report that there was a statistically significant decrease in fastball accuracy after reconstruction; however, the mean pre-operative fastball velocity and curveball movement did not significantly change after surgery [6]. In an analysis of the change in pitching mechanics following UCL reconstruction, Portney et al. [4] do include an evaluation of pitch selection pre- and post-surgery, but this only includes the fastball usage rates between 2008 and 2013.

The purpose of this study is to compare the pre- and post-operative pitch selection of MLB pitchers who received UCL reconstruction between 2015 and 2020, and to assess changes in velocities in the three pitch categories. We hypothesize that pitch selection will favor higher fastball usage following surgery and that velocity will have no significant change.

## Materials and methods

Patient population and demographics

MLB pitchers who underwent UCL reconstruction from 2010 to 2020 were identified using a publicly available database and available media networks. Only pitchers who played at least one full season after surgery and rehabilitation were included in the data collection and analysis. Pitchers who did not play a full major league season before or after surgery were excluded. Data was collected from the last full season played before surgery and the first full season played following return from surgery.

Yearlong statistics

The study primarily analyzed data through publicly available statistics for pitchers. Pitch use percentages and velocity for each pitcher were recorded from Brooks Baseball, a publicly available database that records baseball statistics for MLB players. Earned Run Average (ERA)+ was recorded for each pitcher from Baseball Reference publicly available database that records advanced baseball metrics and accolades for all players.

Pitch selection and analysis

Three pitches were selected for analysis for velocity and usage percentage: fastball, off-speed, and breaking ball. For pitchers with more than three types of pitches, only one of the pitches was counted as an off-speed or breaking pitch. For example, for pitchers with more than one type of breaking ball, only one of the pitches was counted (either slider or curveball). Due to the similarity in pitch type and arm mechanics, sinkers and fastballs were counted as one category. The two pitches were added up for usage and averaged together for velocity.

Pitch control

To learn more about the control of pitchers pre- and post-surgery, instead of looking at strike percentage, we felt that ball percentage was a more accurate portrayal of accuracy. This minimizes swings out of the strike zone and foul balls, and instead puts the focus on how often a pitcher was not within the strike zone that a batter did not swing.

Statistical categories

Data points that were used were the season prior to and after UCL reconstruction. As such, velocity, usage, and ERA+ correspond only to those two corresponding seasons. For example, ERA+ was taken from the year prior to UCL injury and the year following return post-UCL reconstruction surgery. Averages were not used for data points to keep factors such as age-related performance to a minimum. Additionally, all-star game selections were tracked as another component to track before and after performance for top-caliber athletes. Data collection and analysis were performed using Microsoft Excel (Microsoft® Corp., Redmond, WA, USA). Student t-tests were used to compare preoperative and post-operative averages. Pitch usage and velocity were also stratified by top decile, top quartile, bottom quartile, and bottom decile to evaluate for smaller changes given a certain baseline of pitch usage and velocity. Statistical significance was determined based on P <0.05.

## Results

A total of 109 MLB pitchers were identified in our initial search. Eighty-seven MLB pitchers met our study’s criteria and were included in the final analysis. The average number of seasons played in the MLB prior to UCL reconstruction was 4.61, and the average number of seasons played following UCL reconstruction and rehabilitation was 3.67. The average post-operative fastball usage rate was less when compared to the pre-operative group, while the off-speed usage rate increased. However, none of the findings in the usage rates analysis were statistically significant (Table 1). In the velocity analysis, all three pitch groups changed less than 1% from the pre-operative velocity average values (Table 2). ERA+ was found to be smaller in the post-operative group; however, this finding was not significant (pre-ulnar reconstruction (pre-UR) ERA: 109.74, post-ulnar reconstruction (post-UR) ERA: 104.79, P=0.61).

**Table 1:** Pitch Selection and Usage Rates Before and After UCL Reconstruction UCL: ulnar collateral ligament; pre-UR: pre-ulnar reconstruction; post-UR: post-ulnar reconstruction

Pitch Type	Pre-UR	Post-UR	P-value
Fastball	57.86%	54.71%	0.18
Off-Speed	12.26%	14.39%	0.32
Breaking Ball	22.23%	22.89%	0.29

**Table 2:** Pitch Velocity Before and After UCL Reconstruction UCL: ulnar collateral ligament; pre-UR: pre-ulnar reconstruction; post-UR: post-ulnar reconstruction mph: miles per hour

Pitch Type	Pre-UR (mph)	Post-UR (mph)	P-value
Fastball	93.65	93.07	0.26
Off-Speed	86.05	85.82	0.79
Breaking Ball	82.39	82.11	0.80

Quartile analysis

Pitchers in the top decile of pre-surgical fastball percentage had a significant increase in fastball usage following surgery. However, encompassing the entire top quartile, pitchers had a significant decrease in fastball usage following surgery. No other findings in the subgroup analysis were found to be significant (Table 3). The only significant finding in the velocity analysis was that pitchers in the top quartile had a slower fastball velocity following surgery (Table 4).

**Table 3:** Quartile Analysis of Pitch Selection Rates pre-UR: pre-ulnar reconstruction; post-UR: post-ulnar reconstruction

	Fastball			Off-Speed			Breaking Ball		
	Pre-UR	Post-UR	P-value	Pre-UR	Post-UR	P-value	Pre-UR	Post-UR	P-value
Top Decile	69.33%	72.27%	0.02	24.14%	36.37%	0.40	38.42%	37.41%	0.98
Top Quartile	64.42%	62.69%	0.01	15.55%	19.82%	0.12	30.37%	28.86%	0.64
Bottom Quartile	51.23%	45.74%	0.85	4.68%	4.00%	0.19	13.67%	14.41%	0.14
Bottom Decile	44.39%	38.04%	0.93	-*	-*	0.05	7.81%	6.67%	0.44
*Included pitchers with less than 1% usage rate, eliminated due to skewed data									

**Table 4:** Quartile Analysis of Pitch Velocity pre-UR: pre-ulnar reconstruction; post-UR: post-ulnar reconstruction mph: miles per hour

	Fastball			Off-Speed			Breaking Ball		
	Pre-UR (mph)	Post-UR (mph)	P-value	Pre-UR (mph)	Post-UR (mph)	P-value	Pre-UR (mph)	Post-UR (mph)	P-value
Top Decile	96.82	95.84	0.10	89.99	89.26	0.21	87.01	87.67	0.46
Top Quartile	95.51	94.57	0.01	88.10	88.21	0.29	85.94	85.70	0.22
Bottom Quartile	92.19	92.07	0.95	84.55	84.41	0.65	80.16	79.84	0.99
Bottom Decile	91.04	90.73	0.97	82.01	81.52	0.67	76.83	76.24	0.64

## Discussion

UCL injuries are extremely common in MLB pitchers. As most pitchers ultimately require surgical treatment, past studies have reported around an 80% return to play rate [4]. However, the definitions of “return to play” are highly variable. Our study includes a large sample of MLB pitchers who all returned to play for us to compare their post-operative pitch selections to their pre-operative state. Portney et al. [4] reported that there was no significant change in fastball percentage between MLB pitchers who received UCL reconstruction versus those who did not, as well as no significant change in fastball percentage within the group of pitchers who received UCL reconstruction.

Overall, our study suggests a marginal shift from fastball to off-speed and breaking balls following surgery. To our knowledge, only one other study has compared pre-operative and post-operative pitch selection of fastball, off-speed, and breaking balls in MLB pitchers. Peterson et al. [8] also aimed to understand the effects of UCL reconstruction on pitch selection. They reported that there was a significant decline in the percentage of fastballs thrown during the first and second seasons following surgery when compared with the two seasons prior to injury [8]. They also found that there was a statistically significant increase in the mean percentage of curveballs thrown in the second year after surgery [8]. Our findings support this suggested trend, as our results also reported a decrease in fastball usage with an increase in curveball usage in the first season following surgery, although it did not reach statistical significance. Previous studies have reported that pitchers throw more curveballs in the games leading up to UCL injury, and that curveballs place much less torque stress on the medial elbow than fastballs [9]. This would be consistent with Peterson et al.’s findings, as pitchers may be more comfortable throwing more curveballs following surgery to avoid re-injury.

Portney et al. [4] compared pre-operative and post-operative fastball usage rates of pitchers undergoing UCL reconstruction between 2008 and 2013 but did not analyze other pitches. Mayo et al. conducted a retrospective review of MLB pitchers who underwent primary UCL reconstruction between 2009 and 2019 to identify changes in pitch selection, velocity, and spin rate in the 15 games leading up to UCL reconstruction surgery that may predict UCL surgery [10]. They found that there was a significant increase in the percentage of curveballs thrown in the games leading up to UCL injury; however, they did not look at pitch selection after return from surgery [10]. In their velocity analysis, pitchers had a decrease in the velocity of both their fastballs and sliders, which was noted to begin around nine games before injury [10]. Other past studies have analyzed pitch velocity as a potential independent risk factor for UCL injury, although the data is highly variable [11,12]. Although the results of our study indicate a small decrease in pitch velocity following surgery, the findings were not significant. Our data further supports the success of UCL reconstruction surgery as the velocity did not notably in a large sample of MLB pitchers. This data also doesn’t consider age and innings pitched, so given that the velocity does not significantly decline suggests that even after UCL surgery, pitchers are able to return to their pre-operative state. It is reasonable to suggest that pitch velocity would decrease following return from injury to muscle fatigue as well as a change in pitching mechanics. Although some studies have shown that mechanics are similar before and after surgery, a recent biomechanical analysis revealed that fastballs produced the greatest stress across the medial elbow and are significantly more than both changeups and curveballs [13]. This would support the finding that MLB pitchers throw less fastballs after surgery.

Pre- and post-operative performance amongst major league pitchers has been shown to be a mixture of improvement and decline in performance [14-16]. Previous studies have looked at major statistical categories such as wins and losses, ERA, batting average against, walks plus hits per inning pitched, percentage of pitches thrown in the strike zone, innings pitched, percentage fastballs thrown, average fastball velocity, and home runs allowed [14-16]. These statistics can show overall performance, but often whether these before and after competitive levels are due to age-related factors or the ramifications of the procedure itself is difficult to differentiate. Therefore, we have sought to look at behavioral decisions that pitchers make post-procedure with their pitch selection. The results we have collected show that pitchers in total have leaned into their off-speed pitches following surgery with decreased usage of fastballs. This trend of using more off-speed and breaking balls post-surgery was seen in the previous study by Peterson et al. [8].^ ^Furthermore, the decrease in fastball usage has been seen previously [15]. However, whether this pitch selection decision is due to post-surgery hesitancy of fastball usage due to arm stress or a decrease in overall fastball velocity is difficult to elucidate.

Overall, there are plenty of factors that might be contributing to pre- and post-surgical performance metrics. Psychological aspects of injury and rehabilitation might be lending to behavioral decisions in pitchers. Additionally, due to prolonged recovery, year-to-year age factors may still play a larger role in highly competitive performance. An interesting follow-up to our study might be analyzing the cognitive aspects of pitchers pre- and post-surgery, and whether they have the same confidence in their abilities post-UCL reconstruction. Their mindset might be an important factor in their overall performance, their pitch selection, and even their velocity. Additionally, future studies should also analyze individual pitch metrics such as strike and ball percentages and contact and hit percentages to further understand pitch selection in MLB pitchers following UCL injury.

Limitations

This study has several limitations. As a retrospective review, there is always an opportunity for selection bias. The data used for our analysis was collected from publicly available MLB statistics sites, and their accuracy was not verified by an outside source. As this data is strictly collected from MLB pitchers, the findings may not be generalizable to a general population of baseball pitchers. Potential confounders, such as player age, body mass index, and handiness were not considered in this study. The statistical analysis also did not account for other pitch types that may influence the pitch usage results; a majority of the pitchers who utilize certain pitch types regularly use them in a majority of their games. Another limitation is that the data was collected only from the season prior to injury and the initial season returning from surgery and rehabilitation. Limiting the collection to this timeline was done in hopes of decreasing the potential temporal confounding factors such as changes in mechanics or grip, conditioning, and age. Lastly, although we tried to highlight a global trend in performance using the ERA+ advanced metric, these findings are not directly related to any performance results.

## Conclusions

The results of this study show that pitchers tend to throw their secondary and tertiary pitches more often than their fastballs. Our study adds to the literature regarding UCL reconstruction and elite athletic performance levels to help orthopedic surgeons, physiatrists, therapists, and athletes in understanding underlying performance trends. Future studies should focus on the psychological factors that play into a pitcher’s performance.
